# Long Noncoding RNA Expression Signatures of Metastatic Nasopharyngeal Carcinoma and Their Prognostic Value

**DOI:** 10.1155/2015/618924

**Published:** 2015-09-13

**Authors:** Wei Zhang, Lin Wang, Fang Zheng, Ruhai Zou, Changqing Xie, Qiannan Guo, Qian Hu, Jianing Chen, Xing Yang, Herui Yao, Erwei Song, Yanqun Xiang

**Affiliations:** ^1^Breast Tumor Center, Sun Yat-Sen Memorial Hospital, Sun Yat-Sen University, 107 Yanjiang West Road, Guangzhou, Guangdong 510120, China; ^2^Key Laboratory of Malignant Tumor Gene Regulation and Target Therapy of Guangdong Higher Education Institutes, Sun Yat-Sen Memorial Hospital, Sun Yat-Sen University, 107 Yanjiang West Road, Guangzhou, Guangdong 510120, China; ^3^Department of Nasopharyngeal Carcinoma, Sun Yat-Sen University Cancer Center, 651 Dongfeng East Road, Guangzhou, Guangdong 510060, China; ^4^State Key Laboratory of Oncology in Southern China, Guangzhou, China; ^5^Medical Research Center, Sun Yat-Sen Memorial Hospital, Sun Yat-Sen University, 107 Yanjiang West Road, Guangzhou, Guangdong 510120, China; ^6^Department of Ultrasonography, Sun Yat-Sen University Cancer Center, 651 Dongfeng East Road, Guangzhou, Guangdong 510060, China; ^7^Department of Internal Medicine, Brody School of Medicine, Vidant Medical Center, East Carolina University, Greenville, NC, USA; ^8^Department of Oncology, Sun Yat-Sen Memorial Hospital of Sun Yat-Sen University, 107 Yanjiang West Road, Guangzhou, Guangdong 510120, China

## Abstract

Long noncoding RNAs (lncRNAs) have recently been found to play important roles in various cancer types. The elucidation of genome-wide lncRNA expression patterns in metastatic nasopharyngeal carcinoma (NPC) could reveal novel mechanisms underlying NPC carcinogenesis and progression. In this study, lncRNA expression profiling was performed on metastatic and primary NPC tumors, and the differentially expressed lncRNAs between these samples were identified. A total of 33,045 lncRNA probes were generated for our microarray based on authoritative data sources, including RefSeq, UCSC Knowngenes, Ensembl, and related literature. Using these probes, 8,088 lncRNAs were found to be significantly differentially expressed (≥2-fold). To identify the prognostic value of these differentially expressed lncRNAs, four lncRNAs (LOC84740, ENST00000498296, AL359062, and ENST00000438550) were selected; their expression levels were measured in an independent panel of 106 primary NPC samples via QPCR. Among these lncRNAs, ENST00000438550 expression was demonstrated to be significantly correlated with NPC disease progression. A survival analysis showed that a high expression level of ENST00000438550 was an independent indicator of disease progression in NPC patients (*P* = 0.01). In summary, this study may provide novel diagnostic and prognostic biomarkers for NPC, as well as a novel understanding of the mechanism underlying NPC metastasis and potential targets for future treatment.

## 1. Introduction

Nasopharyngeal carcinoma (NPC), a squamous cell carcinoma that occurs in the epithelial lining of the nasopharynx, displays a characteristic geographic and racial distribution worldwide. NPC is a rare malignant tumor in Western countries with an incidence of less than 1/100,000; however, the incidence of NPC was reported to be greater than 20/100,000 in southern China, especially among the Cantonese population living in the central region of Guangdong Province [1, 2]. The histological profile of NPC varies between endemic and nonendemic areas. For example, the tumors from more than 95% of NPC patients in high-incidence areas of China are undifferentiated nonkeratinizing carcinoma, whereas those from patients of Western descent, such as Caucasian, African-American, and Hispanic patients, are predominantly keratinizing squamous cell carcinoma [3–5]. According to the WHO histological profile, NPC among Chinese patients accounts for the majority of nonkeratinizing carcinomas, including 55.9% of the differentiated nonkeratinizing carcinomas and 58.0% of the undifferentiated nonkeratinizing carcinomas. This difference is attributed to the multifactorial etiology of NPC, which includes genetic factors, viral infection, the environment, and dietary habits [5–12]. The cure rate of NPC has improved significantly since the development of radiation technology and chemotherapy. However, distant metastasis remains the primary reason for treatment failure [3, 11, 13]. It is necessary to identify the specific molecular mechanisms that contribute to the pathogenesis and progression of NPC metastasis.

Recent studies suggest that noncoding RNAs (ncRNAs) constitute a large proportion of genome-encoded transcripts [14–16]. There is increasing evidence confirming that ncRNA performs biological functions in both* cis*- and* trans*-gene regulation, especially among higher eukaryotes [16–19]. Due to their functional relevance, ncRNAs have been categorized into housekeeping and regulatory ncRNAs [15]. Long noncoding RNAs (lncRNAs with a length of more than 200 nucleotides) comprise a majority of regulatory ncRNAs [15, 16, 20]. Many lncRNAs are highly conserved and are involved in diverse cellular functions, such as epigenetic regulation [21–23]. lncRNAs have been demonstrated to play crucial roles in dosage compensation, genome imprinting, X chromosome inactivation, chromatin modification, and whole-genome rearrangement [17, 18, 21, 24, 25]. The dysregulated expression of lncRNAs has been identified in a variety of diseases, including different types of cancer [26]; this observation suggests that aberrant lncRNA expression may represent a major contributor to carcinogenesis and cancer progression [17, 27]. For example, HOTAIR and ANRIL act as cancer regulators in carcinogenesis and cancer progression [17, 28]. HOTAIR expression levels increase with clinical stage progression in NPC; NPC patients with high HOTAIR levels have a poor prognosis for overall survival [29]; metastasis-associated lung adenocarcinoma transcript (MALAT-1), PANDA, and ncRNA-DHFR regulate DNA damage, the cell cycle, alternative splicing, and tumor progression [30, 31]. Based on microarray analysis, the H19 gene is strongly expressed in undifferentiated NPC. Furthermore, H19 is highly expressed in an undifferentiated human NPC cell line. H19 plays a role in the differentiation of human NPC cells and the transcriptional silencing of imprinted genes [32]. LINC00312, also named NAG7 (NPC-associated gene 7), is a lincRNA expressed in the cytoplasm of nasopharyngeal epithelial cells. LINC00312 is expressed in 51.4% of NPC samples and 78.4% of noncancerous nasopharyngeal epithelia samples (*P* < 0.001) [33]. Compared with noncancerous nasopharyngeal epithelial tissues, LINC00312 is significantly downregulated in NPC tissues. LINC00312 could be used as a biomarker for NPC metastasis, progression, and prognosis. Based on rematching and reannotation of the existing microarray datasets, five lncRNAs were selected to validate the differential expression of lncRNAs in both primary and recurrent nasopharyngeal carcinoma compared with noncancerous nasopharyngeal epithelia [34]. However, most of the differentially expressed lncRNAs have not been functionally characterized. We suspect that some of these lncRNAs play roles in NPC progression and that some are candidate biomarkers for the diagnosis or prognosis of NPC. The novel molecular mechanisms by which lncRNAs regulate carcinogenesis and metastasis are expected to be elucidated.

In the present study, we performed lncRNA expression profiling on metastatic and primary NPC tumors and identified differentially expressed lncRNAs that could show altered expression prior to or during the invasion-metastasis process. Further investigation validated that the expression level of the lncRNA ENST00000438550 was an independent prognostic marker in NPC patients.

## 2. Materials and Methods

### 2.1. Patients and Tissue Specimens

From July 2010 to November 2012, a total of 110 primary NPC samples and 3 metastatic NPC samples with confirmed pathology were collected from Sun Yat-Sen University Cancer Center. All of the samples were excess discarded tissues from diagnostic procedures. Three NPC metastatic tissue samples were collected via needle biopsy of bone metastatic sites of NPC patients. Among the 110 primary NPC samples, 4 of them were randomly selected for lncRNA microarray analysis. The remaining 106 primary NPC samples underwent QPCR. The tumor tissues from each subject were snap-frozen in liquid nitrogen immediately after biopsy. Written informed consent was obtained from all patients. The research ethics committee of Sun Yat-Sen University Cancer Center approved this study. No patients had received therapy prior to biopsy. The TNM classification of the patients was determined according to the criteria of the American Joint Committee on Cancer (AJCC 7th edition). The detailed clinical information corresponding to the seven NPC patient samples used for microarray analysis is presented in Table S01 in the Supplementary Material available online at http://dx.doi.org/10.1155/2015/618924.

### 2.2. RNA Extraction

Total RNA was extracted from 113 snap-frozen samples using TRIzol reagent (Invitrogen) according to the manufacturer's protocol. The sample quality was evaluated using a Nano Drop ND-1000 spectrophotometer and standard denaturing agarose gel electrophoresis.

### 2.3. Microarray and Computational Analyses

For microarray analysis, the previously prepared total RNA from each sample was purified after rRNA removal (mRNA-ONLY Eukaryotic mRNA Isolation Kit, Epicentre) and then amplified and transcribed into fluorescent cRNA along the entire length of the transcripts without 3′ bias utilizing a random priming method. The labeled cRNAs were hybridized to the Human lncRNA Array v2.0 (8 × 60 K, Arraystar). After washing the slides, the arrays were scanned using the Agilent Scanner G2505C.

Agilent Feature Extraction software (version 11.0.1.1) was used to analyze the acquired array images. Quantile normalization and subsequent data processing were performed using the GeneSpring GX v11.5.1 software package (Agilent Technologies). After quantile normalization of the raw data, lncRNAs in which all 7 samples displayed flags corresponding to Present or Marginal (“All Targets Value”) were selected for further data analysis. The differentially expressed lncRNAs displaying statistical significance between the two groups were identified via Volcano Plot filtering. Finally, hierarchical clustering was performed to elucidate the differentially expressed lncRNA expression profile in the samples.

The experimental protocol was as follows: (1) RNA extraction and RNA QC (described previously); (2) labeling and hybridization (the Agilent Quick Amp Labeling Kit was used for sample labeling and hybridization was performed in Agilent Sure Hyb Hybridization Chambers); (3) data collection and normalization; (4) further data analysis (using Agilent Gene Spring GX v11.5.1 software); and (5) lncRNA classification and subgroup analysis (using home-made scripts). The microarray was performed by KangChen Bio-tech, Shanghai, China.

### 2.4. Quantitative RT-PCR

Real-time PCR was performed using a LightCycler 480 (Roche, Basel, Switzerland). The reactions were performed in triplicate, and the relative expression of lncRNAs (LOC84740, ENST00000498296, AL359062, and ENST00000438550) was normalized to that of the internal control GAPDH. The primer sequences are presented in Supplementary Table S02.

### 2.5. Statistics

Statistical analyses were performed using SPSS version 16.0. Receiver operating characteristic (ROC) curve analysis was used to select the threshold expression levels of the lncRNAs detected via QPCR for disease-free survival (DFS). The survival curves were plotted using the Kaplan-Meier method and were compared using the log-rank test. A multivariate survival analysis was performed using a Cox proportional hazards model (forward). The statistical tests were two-sided, and *P* < 0.05 was considered to be significant.

## 3. Results

### 3.1. Overview of the lncRNA Expression Profiles

Using the lncRNA expression profiles, differentially expressed lncRNAs were determined between the metastatic and primary NPC tumor tissues. The differences in lncRNA expression were evaluated by calculating the normalized fold-change in lncRNA expression between the metastatic/primary tumor (M/T) samples. The selection criterion was a fold-change threshold of 2.0. A positive fold-change indicated upregulation, whereas a negative fold-change indicated downregulation. Log fold-change corresponded to the log2 value of the absolute fold-change. Both the fold-change and the *P* value were normalized. Thousands of lncRNAs were found to be differentially expressed between the metastatic and primary NPC tumors according to UCSC-known gene, Ensemble, RefSeq_NR, H-invDB, NRED, RNAdb, lincRNA, RNAdb, HOX cluster, misc_RNA, UCR, and lncRNAdb.

**Figure 1:**
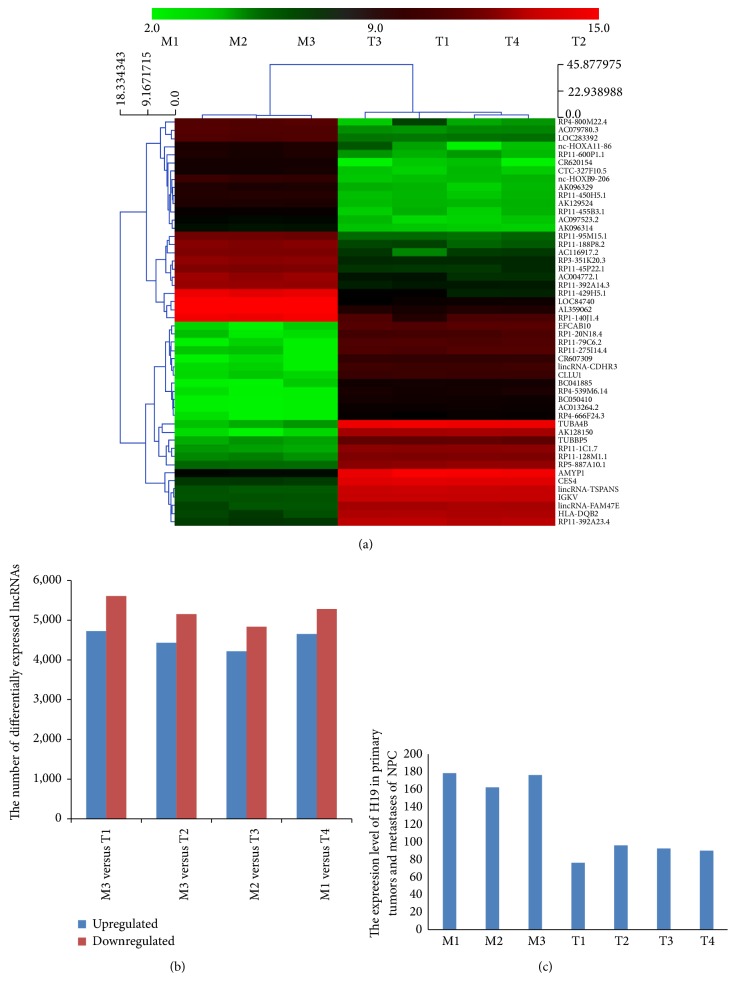
The number of upregulated and downregulated lncRNAs. (a) Hierarchical clustering was performed based on “All Targets Value-lncRNAs.” The results of hierarchical clustering revealed distinct lncRNA expression profiles between the samples. (b) Thousands of lncRNAs were found to be significantly upregulated or downregulated in metastatic NPC tumors compared with primary NPC tumors in seven NPC patients based on microarray analysis. The number of upregulated and downregulated lncRNAs varied between the seven patients. In the four randomly paired M and T groups, downregulated lncRNAs were more common than upregulated lncRNAs. (c) H19 was found to be upregulated in all metastatic samples (*P* < 0.001); the expression levels of H19 were 1.8- to 3-fold higher in the metastatic tumors than in the primary tumors.

A total of 33,045 lncRNA probes were used in our lncRNA microarray. Up to 30,610 lncRNAs were detected in all seven samples (Table S03). Thousands of lncRNAs were found to be differentially expressed, and samples in the same group shared many differentially expressed lncRNAs (Figure 1, Table 1, Table S04). A total of 8,088 lncRNAs were identified to be significantly differentially expressed (≥2-fold) between the metastatic and primary NPC tumors (Table 1, Table S04). Among these, 3,778 lncRNAs were found to be consistently upregulated; 4,310 lncRNAs were downregulated. Additionally, H19 was found to be 2.2-fold upregulated in the metastatic tissue, which could be related to metastasis (Figure 1, Tables S03-S04). CR620154 (log2 fold-change M/T = 94.02) was the most significantly upregulated lncRNA, and TUBA4B (log2 fold-change M/T = −1,364.72) was the most significantly downregulated lncRNA (Table 2).

### 3.2. lncRNA Classification and Subgroup Analysis

**Figure 2:**
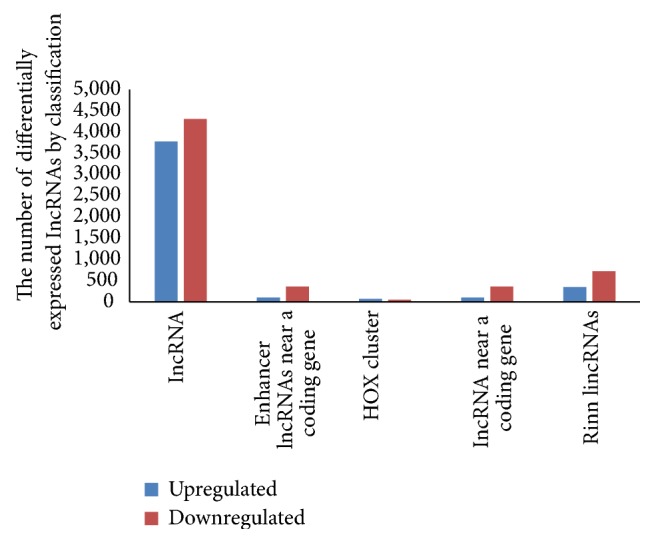
The number of upregulated and downregulated lncRNAs in each subgroup. The lncRNAs were classified into four subgroups based on microarray analysis, including enhancer lncRNAs regulating a nearby coding gene, HOX cluster, lincRNAs regulating a nearby coding gene, and Rinn lincRNAs. The number of lncRNAs that were consistently upregulated or downregulated in the metastatic tumors compared with the primary tumors was calculated for each subgroup.

According to the function and locus of each lncRNA and its association with protein-encoding RNA, Gibb et al. separated lncRNAs into several categories, such as long intronic ncRNAs, antisense RNAs, and promoter-associated long RNAs [35]. In our microarray study, the lncRNAs were classified into four subgroups: enhancer lncRNAs acting on a nearby coding gene, HOX cluster, lncRNAs near a coding gene, and Rinn lincRNAs [23, 36–38]. The expression levels of the lncRNAs in these subgroups were different between the metastatic and primary NPC tumors (Figure 2, Table 1).

**Figure 3:**
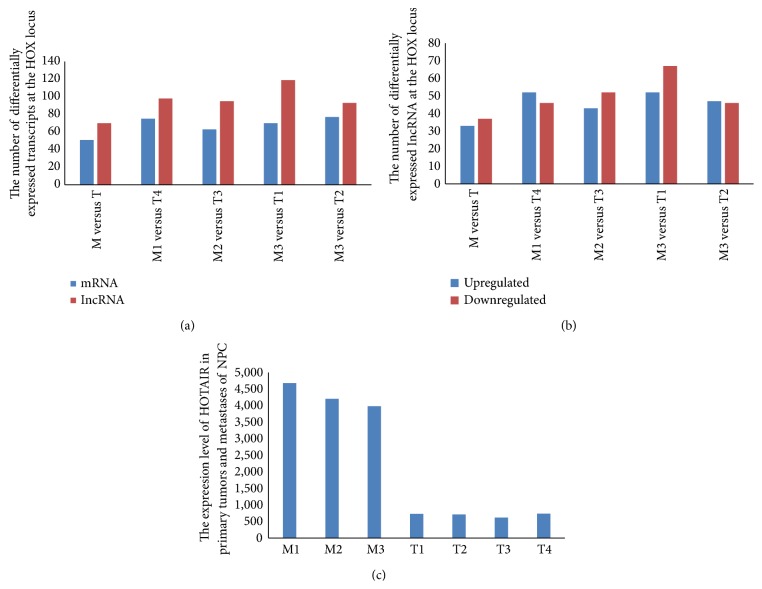
The number of differentially expressed lncRNAs at the HOX locus. (a) The transcripts at the HOX locus varied between the four randomly paired M and T groups. A total of 70 lncRNAs were found to be differentially expressed in the metastatic tissues, and 51 coding transcripts were differentially expressed. (b) Different numbers of lncRNAs were detected in the four randomly paired M and T groups. A total of 33 lncRNAs at the HOX locus were found to be upregulated in all groups, and 37 lncRNAs were downregulated. (c) HOTAIR was found to be upregulated in the metastatic tumor samples from all of the groups (*P* < 0.001); the expression levels of HOTAIR in the metastatic tumors were 4- to 6-fold higher than those in the primary tumors.

In our study, we found that 477 transcribed regions in HOX loci; of these, 257 were ncRNAs and 220 were HOX coding transcripts (Table S05). In the four randomly paired groups, the number of differentially expressed lncRNAs differed, but several lncRNAs displayed similar changes in expression. Compared with the NPC primary tumors, 70 lncRNAs were found to be differentially expressed in metastatic tissues; 51 coding transcripts were differentially expressed (Table S06, Figure 3(a)). According to the comparative analysis of the four randomly paired groups, 33 lncRNAs were upregulated and 37 lncRNAs were downregulated in the metastatic NPC samples compared with the primary NPC tumor samples (Figure 3(b)). Interestingly, HOTAIR, a known regulatory lncRNA located at the HOX locus, was among the 33 upregulated lncRNAs (Figure 3(c)). HOTAIR has been demonstrated to be an oncogene to modulate the metastasis of breast cancer and NPC [17, 39].

**Figure 4:**
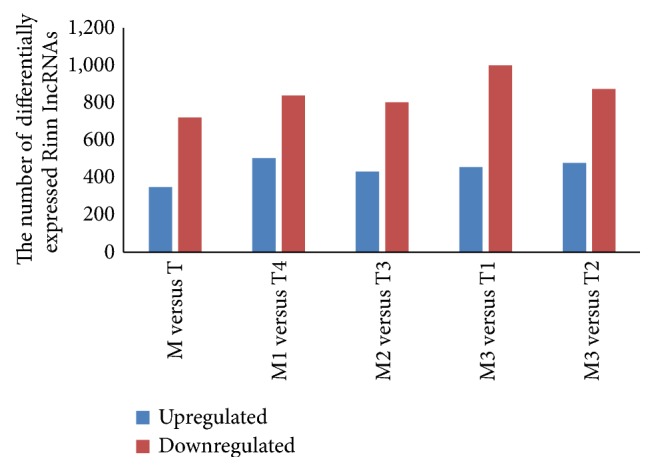
The number of differentially expressed Rinn lincRNAs. Rinn lincRNAs are a type of lincRNAs termed based on studies by Rinn. A total of 4,199 Rinn lincRNAs were detected in our microarray analysis. The number of downregulated Rinn lincRNAs was greater than the number of upregulated Rinn lincRNAs. According to the expression levels of all detected Rinn lincRNAs in metastatic and primary NPC tumors, 348 of these lncRNAs displayed consistent upregulation and 721 of these lncRNAs displayed consistent downregulation in the four randomly paired M and T groups.

Rinn lincRNAs, a type of lincRNAs identified by Rinn, were also detected in our study [23, 38]. A total of 4,199 Rinn lincRNAs were detected in our microarray (Table S07). The number of upregulated and downregulated Rinn lincRNAs varied between the seven patients. A total of 1,069 Rinn lincRNAs were found to be differentially expressed between the patient samples (Figure 4, Table S08). As shown in Figure 4, the downregulation of the lncRNAs was more common than the upregulation. Among the four paired samples, we found 348 lncRNAs that were consistently upregulated and 721 lncRNAs that were consistently downregulated. The consistently dysregulated lncRNAs in the four groups may function as oncogenes or tumor suppressor genes; this merits further investigation.

**Figure 5:**
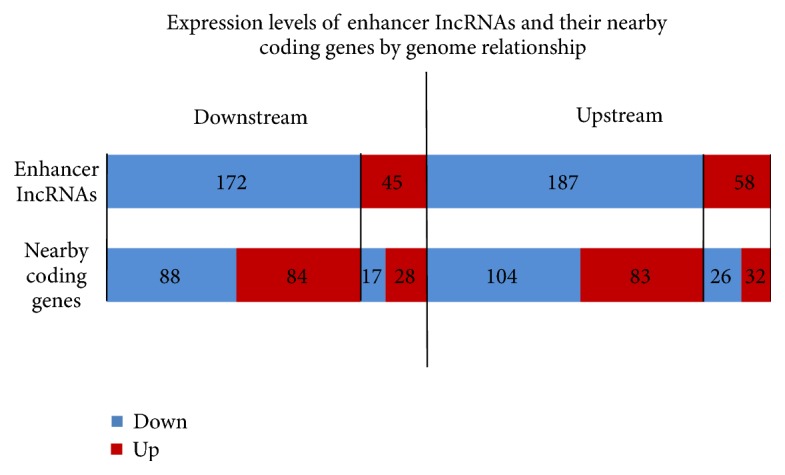
Expression levels of enhancer lncRNAs and their nearby coding genes based on genome relationship analysis. A total of 245 enhancer lncRNAs upstream of their nearby coding genes were differentially expressed; 58 of these enhancer lncRNAs were upregulated and 187 were downregulated. Additionally, 217 enhancer lncRNAs downstream of their nearby coding genes were differentially expressed; 45 of these enhancer lncRNAs were upregulated and 172 were downregulated. Some of the nearby coding genes displayed consistent upregulation or downregulation in concert with that of their corresponding enhancer lncRNAs, whereas other nearby coding genes displayed opposite differences.

Enhancer lncRNAs acting on a nearby coding gene were first found in human cell lines [37]. The present study revealed an unanticipated role of this subgroup of lncRNAs in the activation of critical development and differentiation regulators. In this study, many enhancer lncRNAs were found to display increased or decreased expression in M/T. Enhancer lncRNA profiling contained the profiling data of all lncRNAs displaying enhancer-like function (Table S09). A total of 1,598 enhancer lncRNAs were detected, of which 468 were differentially expressed. The differentially expressed enhancer lncRNAs and their nearby coding genes (distance < 300 kb) are presented in Table S10. As shown in Figure 5, the enhancer lncRNAs were located either upstream or downstream of the coding genes. Some of the enhancer lncRNAs shared the same change in expression with their nearby coding genes, while the others displayed the opposite changes; this was helpful for the identification of functional enhancer lncRNAs.

We performed a further analysis of the lincRNA profiles to identify additional potential regulatory lncRNAs and their target genes among the lincRNAs. The differentially expressed lincRNAs and nearby coding gene pairs (distance < 300 kb) are provided in Table S11 (*P* < 0.05).

### 3.3. Real-Time Quantitative PCR Validation

**Figure 6:**
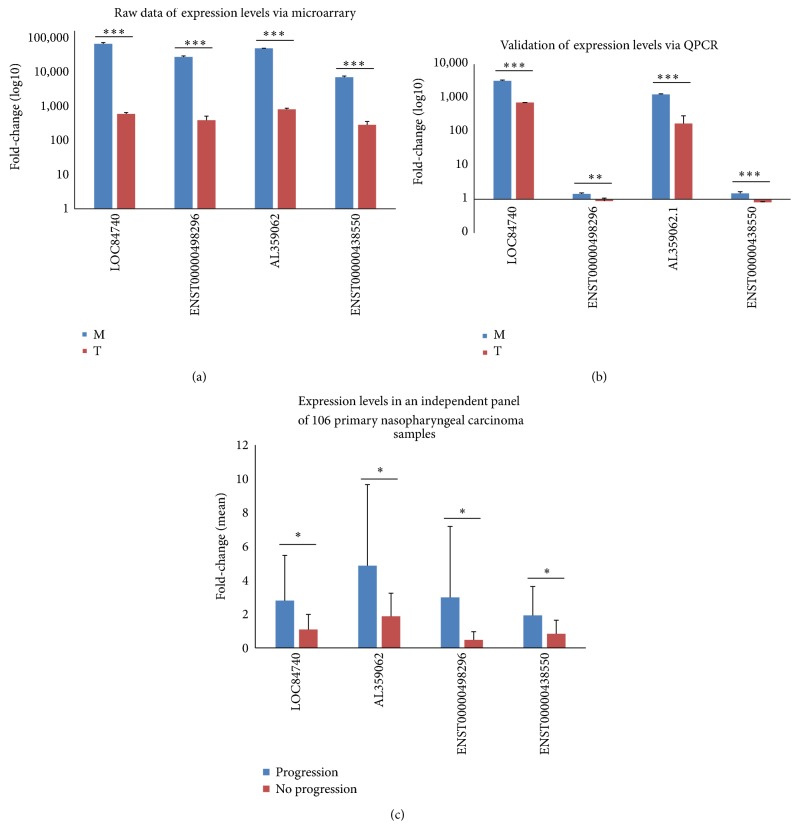
Real-time quantitative PCR validation. (a) Raw data of the expression levels of the four lncRNAs (LOC84740, ENST00000498296, AL359062, and ENST00000438550) based on microarray analysis. All four of these lncRNAs displayed a high basal expression level in metastatic and primary NPC tumors, but their expression levels significantly differed between the two groups. ^*∗∗∗*^ *P* < 0.001. (b) Validation of the microarray data. All four lncRNAs (LOC84740, ENST00000498296, AL359062, and ENST00000438550) were differentially expressed in the metastatic and primary NPC tumors based on microarray analysis, which was validated via QPCR using the same tissues. The validation results of the four lncRNAs indicated that the microarray data strongly correlated with the QPCR results. ^*∗∗∗*^ *P* < 0.001 and ^*∗∗*^ *P* < 0.01. (c) The expression levels of four lncRNAs (LOC84740, ENST00000498296, AL359062, and ENST00000438550) were measured in an independent panel of 106 primary NPC samples via QPCR. These lncRNAs displayed higher expression levels in NPC primary tumors with progression than in those without progression. ^*∗*^ *P* < 0.05.

Based on this microarray analysis and according to the baseline and fold-change in the expression levels, four different lncRNA members (LOC84740, ENST00000498296, AL359062, and ENST00000438550) were selected to verify their expression levels via QPCR. The results revealed strong consistency among the QPCR results and the microarray data (Figures 6(a)-6(b)). Additionally, the expression levels of the four lncRNAs (LOC84740, ENST00000498296, AL359062, and ENST00000438550) were measured in an independent panel of 106 primary NPC samples via QPCR; however, the sample from one patient did not show expression of LOC84740 (Figure 6(c), Table 3). The clinicopathological characteristics of these 106 patients and the associations between these characteristics and the expression levels of LOC84740, ENST00000498296, AL359062, and ENST00000438550 are presented in Table 3. According to their respective ROC curves, the fold-change cutoff points in the expression thresholds for LOC84740, ENST00000498296, AL359062, and ENST00000438550 were 5.54, 0.37, 3.76, and 0.43, respectively. The expression levels of the lncRNAs were categorized into high and low levels accordingly. A high ENST00000438550 expression level was associated with disease progression among NPC patients (*P* = 0.01).

### 3.4. Prognosis of NPC Patients Displaying Differentially Expressed lncRNAs

**Figure 7:**
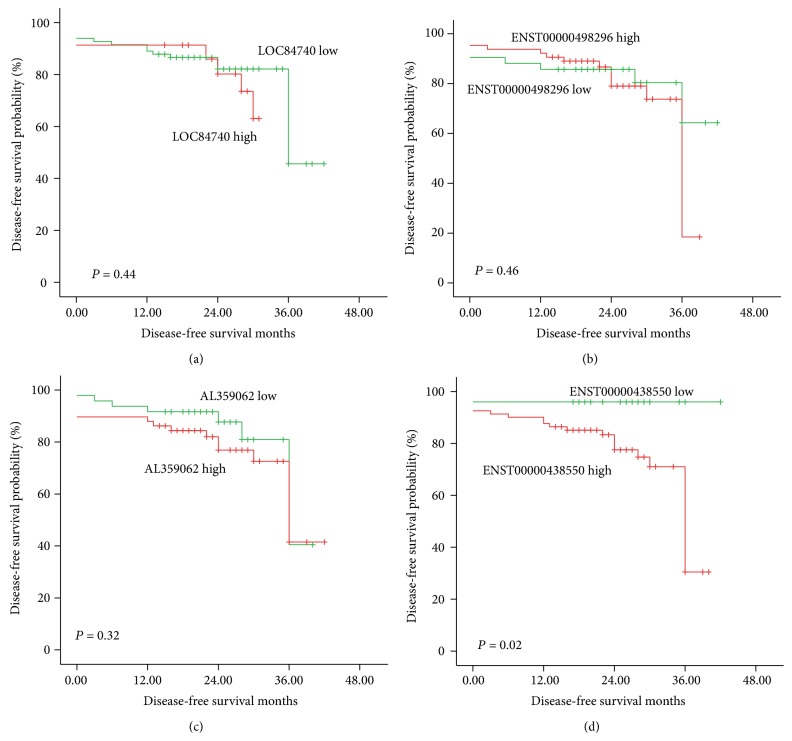
DFS. The expression level of ENST00000438550 correlated with disease progression in NPC patients; those displaying a high ENST00000438550 expression level experienced a significantly shorter DFS (d). However, the expression levels of LOC84740, ENST00000498296, and AL359062 were not correlated with the DFS of the NPC patients ((a)–(c)).

To further confirm the prognostic value of these lncRNAs for NPC, the DFS of the four lncRNAs was analyzed. Among them, only ENST00000438550 was a significant predictor of disease progression in NPC patients (3-year DFS of 96% and 73% for the low and high level groups, respectively, *P* = 0.02, Figure 7). A multivariate analysis was performed using the COX proportional hazards model to analyze the prognostic values of age, gender, T classification, N classification, and the expression levels of LOC84740, ENST00000498296, AL359062, and ENST00000438550. The results revealed that only the expression level of ENST00000438550 was an independent prognostic indicator of disease progression in NPC patients (*Chi* square = 6.64, *P* = 0.01). These results suggested that ENST00000438550 could serve as a prognostic marker in NPC patients.

## 4. Discussion

The present study was the first to demonstrate that lncRNAs are differentially expressed between metastatic and primary NPC tumors. There have been no previous reports describing lncRNA expression profiles of NPC samples that also performed a differential expression analysis. Furthermore, this study was the first to demonstrate that a high expression level of ENST00000438550 is an independent indicator of disease progression in NPC patients.

Epstein-Barr virus (EBV) plays very important roles in the carcinogenesis of NPC. EBV exhibits tumorigenic potential due to a unique set of latent genes. Latent membrane protein-1 (LMP1) is the principal oncogene, and its expression level is a prognostic marker of NPC [40]. With the development of microarray technology, novel potential therapeutic targets as well as diagnostic and prognostic biomarkers have been identified based on gene expression array analyses. lncRNA expression array analysis has been used in oncology studies in recent years. A variety of lncRNAs, including ANRIL, MEG3 and HULC, either promote or suppress the development of cancer [41–44]. Among these, XIST is a well-known imprinted lncRNA that is abnormally expressed in ovarian and breast cancers [45, 46]. MALAT-1 was the first lncRNA that was found to be associated with high metastatic potential and poor patient prognosis in non-small-cell lung cancer patients [47]. MALAT-1 is also upregulated in other human cancers, such as breast cancer, prostate cancer, colorectal cancer, liver cancer, and uterine cancer [48–51]. These findings imply an association between lncRNAs and carcinogenesis.

The altered expression of many genes has been reported to be associated with the development of NPC [52, 53]. HOTAIR, a lincRNA in the mammalian HOXC locus, was the first lincRNA that was found to be systematically dysregulated during breast cancer progression via microarray analysis [17]. Further evidence indicates that HOTAIR reprograms the chromatin state to promote cancer metastasis and primary tumor growth* in vivo* [17]. HOTAIR has been proposed as a putative biomarker for metastasis of human malignant tumors, and it is a powerful predictor of eventual metastasis and death [17, 29]. HOTAIR is aberrantly expressed in several carcinomas, including NPC [29, 39, 54–56]. HOTAIR is upregulated in cases of NPC at more advanced clinical stage and with increased lymph node tumor burden [29]. In our study, HOTAIR was also consistently upregulated in metastatic samples, indicating that increased HOTAIR expression is associated with the progression and development of NPC. H19, another imprinted lncRNA gene with high expression levels during vertebrate embryo development, is downregulated in most tissues shortly after birth [57]. Its loss of imprinting and aberrant expression has been detected in various cancers and has been demonstrated to play a key role in oncogenesis and tumor suppression [18, 32, 58–61]. H19 expression is induced by hypermethylation of its promoter region. H19 is significantly upregulated in the undifferentiated human NPC cell line CNE-2, but it is not expressed in well-differentiated human HK1 NPC cells [32]. Our study observed that H19 expression was upregulated in metastatic NPC tumors compared with primary NPC tumors; this result suggests that H19 expression is related to NPC progression.

lincRNA LINC00312 is significantly downregulated in NPC tissues compared with noncancerous nasopharyngeal epithelial tissues as assessed by a NPC tissue microarray [33]. However, we did not find LINC00312 in our differentially expressed lncRNAs. The expression of LINC00312 decreased with NPC progression. In addition, only half of the NPC samples express LINC00312, and the number of samples used in our microarray study is limited. Five lncRNAs (lnc-C22orf32-1, lnc-TLR4-1, lnc-BCL2L11-3, lnc-AL355149.1-1, and lnc-ZNF674-1) were differentially expressed in NPC compared with normal nasopharyngeal epithelial tissues in the microarray data set GSE12452 [34]. Four of these lncRNAs (lnc-C22orf32-1, lncTLR4-1, lnc-AL355149.1-1, and lnc-ZNF674-1) demonstrated significant expression differences between primary NPC and normal nasopharyngeal samples via QPCR. Only lnc-BCL2L11-3 was upregulated in the recurrent NPC tissues compared with the paired normal tissues. lnc-AL355149.1-1 and lnc-ZNF674-1 were downregulated compared with primary NPC. Unfortunately, none of the five lncRNAs were identified among the differentially expressed lncRNAs based on our microarray data. Our research focuses on NPC metastasis, and the expression of the five lncRNAs varied during carcinogenesis and recurrence.

Based on this microarray analysis and according to the baseline and fold-change in the expression levels, four lncRNAs (LOC84740, ENST00000498296, AL359062, and ENST00000438550) were selected to validate the microarray results and to evaluate their roles as biomarkers in NPC patients. Consistent with the microarray results, the four lncRNAs were differentially expressed based on QPCR. To further illustrate the relationship between these four lncRNAs and NPC prognosis, we analyzed the expression levels of these four lncRNAs via QPCR and evaluated their potential values as prognostic indicators of NPC. We found that, among these four lncRNAs, only ENST00000438550 was an independent prognostic indicator of disease progression in NPC patients. The expression level of ENST00000438550 was negatively correlated with the prognosis of NPC patients; this suggests that elucidating the role of ENST00000438550 in NPC progression may contribute to understanding of the mechanism of NPC metastasis.

## 5. Conclusion

To the best of our knowledge, few differentially expressed lncRNAs have been reported in NPC, and this is the first report elucidating the lncRNA expression profiles of metastatic and primary NPC tumor tissues. Further investigation is required in the search for additional functional lncRNAs in NPC. This study has limitations, including the limited sample number for microarray analysis, which was partially due to the difficulty in conducting bone metastases biopsies. In brief, our finding provides new insights into understanding NPC. lncRNAs may underlie novel mechanisms of NPC and may represent potential targets for NPC treatment and prognostic factors for NPC, which are expected to be elucidated in the near future.

## Supplementary Material

Table S01. The clinical information corresponding to the seven NPC patient samples used for microarray analysis.
Table S02. Four different lncRNA members (LOC84740, ENST00000498296, AL359062, and ENST00000438550) were selected to verify their expression levels via QPCR. This table supply the primer sequences used for Real-time PCR of the four lncRNAs.
Table S03. lncRNA Expression Profiling Data. A total of 33,045 lncRNA probes were used in our lncRNA microarray. Up to 30,610 lncRNAs were detected in all seven samples.
Table S04. Differentially Expressed lncRNAs. A total of 8,088 lncRNAs were identified to be significantly differentially expressed(≥2-fold) between the metastatic and primary NPC tumors in the microarray. Among these, 3,778 lncRNAs were found to be consistently upregulated; 4,310 lncRNAs were downregulated.
Table S05. HOX cluster profiling. In this study, we detected the transcripts in HOX loci, 477 transcribed regions were found. Among them, 257 were ncRNAs and 220 were HOX coding transcripts.
Table S06. Differentially expressed IncRNAs at HOX locus. In the four randomly paired groups, the number of differentially expressed lncRNAs differed, but several lncRNAs displayed similar changes in expression. Compared with the NPC primary tumors, 70 lncRNAs were found to be differentially expressed in metastatic tissues; 51 coding transcripts were differentially expressed.
Table S07. Rinn lincRNA expression profiles. A total of 4,199 Rinn lincRNAs were detected in this microarray analysis.
Table S08. Differentially expressed Rinn lincRNAs. A total of 1,069 Rinn lincRNAs were found to be differentially expressed.
Table S09. Enhancer lncRNAs profiles. A total of 1,598 enhancer lncRNAs were detected, of which 468 lncRNAs were differentially expressed.
Table S10. Data for enhancer lncRNAs regulating a nearby coding gene. The differentially expressed enhancer lncRNAs and their nearby coding genes (distance < 300 kb) were presented.
Table S11. Data for lincRNAs regulating a nearby coding gene. The differentially expressed lincRNAs and their nearby coding gene pairs (distance < 300 kb) were presented.

## Figures and Tables

**Table 1:** Summary of the microarray data for the metastatic and primary NPC tumors.

Gene type	RNA expression	Fold-change (*n*)				Differentially expressed RNAs (*n*)
		>20	10–20	2–10	Total	
lncRNA	Upregulated	65	191	3,522	3,778	8,088
	Downregulated	198	225	3,887	4,310	

Enhancer lncRNAs regulating a nearby coding gene	Upregulated	4	5	94	103	462
	Downregulated	17	20	322	359	

HOX cluster	Upregulated	15	5	51	71	121
	Downregulated	1	3	46	50	

lncRNAs regulating a nearby coding gene	Upregulated	4	5	94	103	462
	Downregulated	17	20	322	359	

Rinn lincRNAs	Upregulated	6	14	328	348	1,069
	Downregulated	33	28	660	721	

NPC: nasopharyngeal carcinoma; lncRNA: long noncoding RNA; lincRNA: long intergenic noncoding RNA.

**Table 2:** A collection of significantly differentially expressed lncRNAs detected via microarray analysis in seven NPC patients.

Upregulated		Downregulated	
lncRNA	log2 fold-change (M/T)	lncRNA	log2 fold-change (M/T)
CR620154	94.02	TUBA4B	1,364.72
LOC84740	92.86	AK128150	1,120.20
nc-HOXB9-206	87.55	RP11-79C6.2	303.82
RP4-800M22.4	67.25	EFCAB10	274.84
RP11-450H5.1	65.75	RP11-1C1.7	208.11
RP11-429H5.1	64.15	RP11-275I14.4	206.67
CTC-327F10.5	62.57	CR607309	197.51
AK096329	58.19	RP1-20N18.4	196.02
AC079780.3	55.47	lincRNA-CDHR3	195.01
AK129524	53.04	BC041885	141.04
AL359062	50.10	lincRNA-TSPAN8	137.80
RP11-95M15.1	46.94	BC050410	131.80
RP11-455B3.1	45.48	RP4-539M6.14	130.89
RP11-188P8.2	41.74	IGKV	125.27
AC097523.2	41.72	AC013264.2	122.93
nc-HOXA11-86	41.48	CES4	120.27
RP11-600P1.1	38.72	CLLU1	108.35
LOC283392	33.97	TUBBP5	108.01
AK096314	33.87	RP11-128M1.1	105.58
AC116917.2	33.61	RP4-666F24.3	100.17

NPC: nasopharyngeal carcinoma; M: metastatic NPC tissue; T: primary NPC tissue.

False discovery rate (FDR) < 0.1%, *P* < 0.01.

**Table 3:** The clinicopathological characteristics and their association with the expression levels of four lncRNAs (LOC84740, ENST00000498296, AL359062, and ENST00000438550) in NPC patients.

	LOC84740 (*n* = 105)			ENST00000498296 (*n* = 106)			AL359062 (*n* = 106)			ENST00000438550 (*n* = 106)		
	L	H	*P*	L	H	*P*	L	H	*P*	L	H	*P*
Age (years)												
<50	53	14	0.81	25	43	0.54	34	34	0.23	12	56	0.06
≥50	29	9		17	21		14	24		13	25	
Gender												
Male	66	15	0.15	32	49	1.00	37	44	0.82	11	70	0.00
Female	15	8		9	15		10	14		14	10	
Histological type												
D	2	0	0.00	0	2	0.52	1	1	1.00	1	1	0.42
U	80	23		42	62		47	57		24	80	
T classification												
T1-2	7	0	0.34	4	3	0.43	5	2	0.24	1	6	1.00
T3-4	75	23		38	61		43	56		24	75	
N classification												
N0-1	51	16	0.63	26	41	0.84	28	39	0.42	15	52	0.81
N2-3	31	7		16	23		20	19		10	29	
Distant metastasis												
No	73	18	0.18	36	56	0.78	44	48	0.25	24	68	0.18
Yes	9	5		6	8		4	10		1	13	
Local-regional relapse												
No	74	21	1.00	40	56	0.31	45	51	0.34	25	71	0.11
Yes	8	2		2	8		3	7		0	10	
Disease progression												
No	65	17	0.58	34	49	0.64	41	42	0.16	24	59	0.01
Yes	17	6		8	15		7	16		1	22	

L: low level; H: high level; *P*: *P* value; D: differentiated nonkeratinized carcinoma; U: undifferentiated nonkeratinized carcinoma.
